# Multiple Peripheral Schwannomas

**Published:** 2018-03-19

**Authors:** Cara M. Barber, Matthew P. Fahrenkopf, Nicholas S. Adams, Steven C. Naum

**Affiliations:** ^a^Michigan State University College of Human Medicine, Grand Rapids; ^b^Spectrum Health/Michigan State University Plastic Surgery Residency, Grand Rapids; ^c^Orthopedic Associates of Michigan, Grand Rapids

**Keywords:** schwannomatosis, nerve sheath tumor, median nerve, schwannoma, upper extremity

## DESCRIPTION

**Figure 1:**
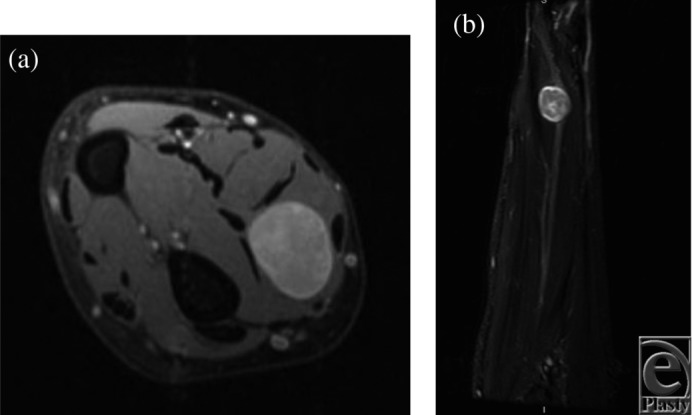
Axial T2 (a) and coronal STIR (b) MRI of the right distal forearm showing a well-circumscribed mass with hyperintense signal. The median nerve is seen in the coronal view with displacement of adjacent tissues.

**Figure 2:**
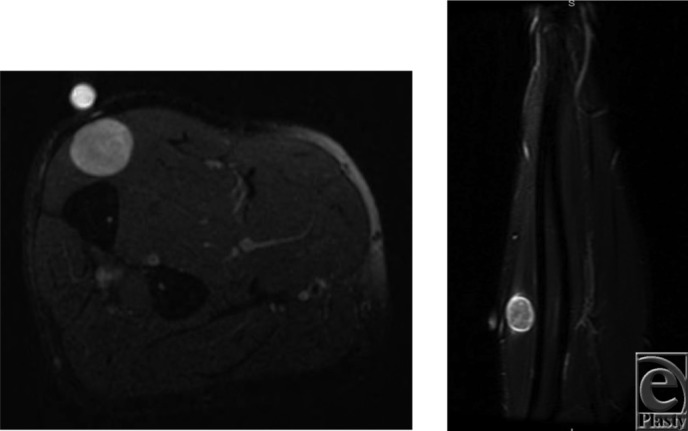
Axial T2 (a) and coronal STIR (b) MRI of the right proximal forearm with a hyperintense, well-circumscribed mass within the flexor digitorum profundus.

A 57 year-old-man presented to the clinic with painless swelling in his right distal volar forearm. His medical history was significant for a previously resected autistic neuroma. Magnetic resonance imaging (MRI) was performed. Two schwannomas were present involving the median nerve (Figs 1*a* and 1*b*) and a muscular branch of the ulnar nerve to the flexor digitorum profundus (Figs 2*a* and 2*b*).

## QUESTIONS

1. How do schwannomas typically present?
2. Who is at risk of developing schwannomas?
3. How are schwannomas treated?
4. What is the difference in outcomes between neurofibromas and schwannomas?

## DISCUSSION

**Figure 3:**
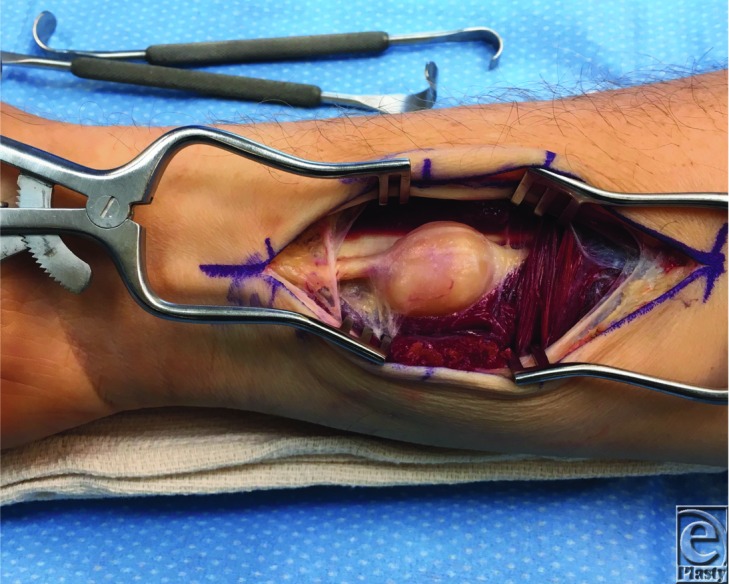
Schwannoma involving the median nerve in the distal forearm. Note the intraneural location of the tumor.

Schwannomas are benign tumors that develop from the peripheral myelin-producing cells, the Schwann cells. Schwannomas can present in various locations throughout the peripheral nervous system but are most commonly found in the head and neck, accounting for up to 45% of all schwannomas.^1^ Patients presenting with schwannomas typically complain of chronic neuropathic pain due to the compression of the adjacent nerve. The presence of a noticeable mass is also frequently seen with peripheral tumors.^2^ The diagnosis of schwannoma is made through a thorough history and physical examination and confirmed with imaging, including computed tomography and MRI, and tissue biopsy.^3^ A key examination finding of these tumors is mobility with transverse motion but not in the longitudinal axis. This is due to their nested, intraneural location (Fig 3).^3^ A differential diagnosis should also include neurofibroma, ganglion, sarcoma, lipoma, and xanthomas.^3^

Schwannomas most commonly present within the third to sixth decades of life.^3^ Most are idiopathic; however, they can be associated with genetic disorders. Schwannomatosis is the development of multiple Schwann cell tumors in the periphery.^4^ Schwannomatosis has been shown to have an autosomal dominant inheritance pattern with incomplete penetrance. It is most closely associated with mutations in tumor suppressor genes, including *SMARCB1* or *INI1*.^4^ Schwannomatosis falls under the umbrella of neurofibromatosis (NF), which has 3 distinct disorders: NF type 1 (NF1), NF type 2 (NF2), and schwannomatosis.^4^ Neurofibromas are benign tumors of the nerve sheath and have been previously understood to be isolated from schwannoma formation.^4^ Both NF and schwannomatosis are seen equally in all races and genders. In addition, it is important to note that one type cannot turn into another, although NF2 and schwannomatosis are found on the same chromosome, chromosome 22.^4^ The patient did not carry a diagnosis of NF2 prior to the identification of these tumors but met criteria for NF2 due to his history of acoustic neuroma and 2 additional NF-related tumors (schwannomas).

Management of these tumors is based mainly on the severity of symptoms. For those with symptomatic schwannomas, the treatment is surgical. This often involves intraneural dissection requiring meticulous technique to maintain function of the involved nerve.^5^ If the tumor capsule is densely adherent to the parent nerve, it is left behind. Currently, there are no medical treatments of schwannomas.

**Figure 4:**
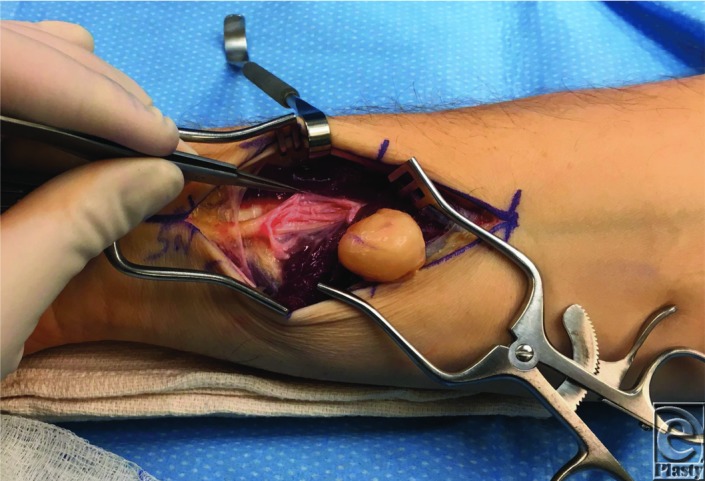
Schwannoma removed from the median nerve in the distal forearm. With careful dissection, the encapsulated tumor can be removed from the surrounding nerve fascicles.

The surgical interventions for neurofibroma and schwannoma vary slightly, as do their outcomes for nerve preservation and recurrence.^6^ Resolution of schwannomas is quite successful, where surgical removal involves little or no injury to the parent nerve.^7^ Recurrence of schwannomas after total removal is rare.^8^ This is due to the structural difference between neurofibromas and schwannomas, the latter being encapsulated and easier to remove with meticulous technique (Fig 4).^8^ On the contrary, neurofibromas are often intimately integrated into the nerve fibers and have a greater potential for lasting nerve damage following resection.^8^ The most common postoperative complication is paresthesia.

In summary, schwannomas are uncommon in the upper extremity, accounting for 0.1% to 0.3% of all hand tumors. When present, they may be associated with genetic abnormalities. There is currently no medical treatment of these benign tumors, but surgical outcomes are very positive.^3^^,^^4^
